# Molecular Evidence of Chlamydia trachomatis Infection and Its Relation to Miscarriage

**DOI:** 10.22074/ijfs.2018.5184

**Published:** 2018-03-18

**Authors:** Sahar Bagheri, Rasoul Roghanian, Naser Golbang, Pouran Golbang, Mohammad Hossein Nasr Esfahani

**Affiliations:** 1 Department of Biology, Faculty of Science, University of Isfahan, Isfahan, Iran; 2 Department of Obstetrics and Gynecology, Emam Khomeini Hospital, Falavarjan, Isfahan, Iran; 3 Departmen of Reproductive Biotechnology, Reproductive Biomedicine Research Centre, Royan Institute for Biotechnology, ACECR, Isfahan, Iran

**Keywords:** Chlamydia trachomatis, Enzyme-Linked Immunosorbent Assay, Miscarriage, Polymerase Chain Reaction

## Abstract

**Background:**

Chlamydia trachomatis (CT) infection is the most common sexually transmitted disease in the world that can persist and also ascend in the genital tract. This intracellular and silent infection is related to some adverse pregnancy outcomes, such as miscarriage. The aims of this study were to explore the best CT screening tests using blood and vaginal samples and to investigate the correlation between CT infection and the incidence of miscarriage.

**Materials and Methods:**

This case-control study was done in October 2013 through June 2014, using purposive sampling from 157 female participants with or without a history of miscarriage. The samples were taken after each participant had signed a letter of consent and had completed a questionnaire. To achieve the objectives of this study, polymerase chain reaction (PCR) and enzyme-linked immunosorbent assay (ELISA) tests were performed on vaginal swabs and blood samples, respectively.

**Results:**

PCR results showed a significantly higher CT infection rate in the miscarriage group compared to the control group (11.3 vs. 0%, P=0.007). Anti-CT IgG and IgA antibodies were found in 4.2 and 2.1% of cases in the miscarriage group, and in 1.7 and 6.7% of cases in the control group, respectively (P>0.05). Despite lower humoral responses in this study, positive samples were detected only by one of the following techniques; PCR, ELISA IgA and ELISA IgG. It also should be noted that PCR worked best in terms of detection.

**Conclusion:**

Based on the obtained data, there is a strong association between molecular evidence of CT infection and miscarriage. A higher rate of CT detection in molecular tests compared to serological assays suggests that PCR could be used as the first-choice assay for detection of C. trachomatis. However, the importance of serological tests in detecting potential past CT infection or upper genital infection not amenable to sampling is undeniable.

## Introduction

Although *chlamydiaewere* discovered in 1907, chlamydial disease was known for centuries before that. Chlamydia trachomatis (CT) is a Gram-negative, non- motile and obligate intracellular bacterium, which causes one of the most prevalent sexually transmitted diseases called chlamydia (1). The World Health Organisation estimated that compared to the year 2005, 131 million new cases of urogenital CT infection have occurred in women and men aged 15-49 years globally in the year 2012 (2). 

This genital infection can result in adverse reproductive outcomes such as infertility, premature delivery, ectopic pregnancy, low birth weight, and miscarriage (3). Despite significant progress in medical sciences, many miscarriages still occur. Miscarriage is the most common sequela of pregnancy, which is defined as pregnancy loss before the 24^th^ week of gestation (4). Nonetheless, in most cases, the causes of miscarriage are unknown. Nonaka et al. (5) have reported that prevalence of chromosomal aberrations in patients with a history of recurrent spontaneous abortion is less than those who have sporadic miscarriage. Also, in women with recurrent spontaneous abortion, 25- 32% of conception products have abnormal karyotypes. On the one hand, chromosomal aberrations are observed in approximately 50% of early miscarriages. On the other hand, infections have been attributed to 15% of early miscarriages and 66% of late miscarriages (6). 

Enzyme linked immunosorbent assay (ELISA) and polymerase chain reaction (PCR) are two common methods for detection of CT. In 2003-2006, a group in Poland evaluated the frequency of CT infection in women suffering from spontaneous miscarriage by PCR and ELISA IgG and IgA (7). Also, the same serologic and molecular tests were used to investigate whether CT is related to miscarriage in Switzerland (8). Results of a serologic study for diagnosis of CT in sub fertile women suggests that MOMP-based (major outer membrane protein of CT used as antigen) ELISA is equally suitable, if not slightly better, than micro-immunofluorescence assays in terms of sensitivity and specificity (9). For detection of CT in laboratories, Nucleic Acid Amplification tests have been reported to function better than other available tests because of their sensitivity and specificity (8). Since the method of choice for detection of CT is ELISA in most clinics, this study was done to evaluate the validity of this method to improve the screening program for detection of this infection. Moreover, the correlation between CT infection and the incidence of miscarriage was investigated.

## Materials and Methods

This study was a case-control study and samples were collected starting in October 2013 through June 2014. The sample size was calculated with regard to the reported prevalence of CT (10), 95% confidence interval and 80% power. The control group comprised 60 pregnant women without any miscarriage history, ranging from 20 to 40 years of age (mean 27.85 ± 5.14 years), who attended a pregnancy assessment unit. The miscarriage groups included 55 women with 1-2 and 42 women with =3 miscarriages, ranging from 19 to 45 years of age (mean 30.88 ± 5.9 years), who were referred to a Fertility Centre in Isfahan, Iran. In the miscarriage group, samples were taken after the last miscarriage (4-24 weeks of gestation) and termination of bleeding. 

All participants were married and had one sex partner. Local Ethical Committee approval and participants’ consent were obtained. A questionnaire containing demographic information, anti-biotherapy history, and previous adverse pregnancy outcome was completed by participants. The criteria for participant selection were no use of any chlamydia-related antibiotics during the last three months, no bleeding, and submitting a completed questionnaire. In order to exclude cases who most likely had genetic problems, questions regarding possible products of conception with congenital malformation and developmental delay, past karyotype tests, and a history of infertility and genetic disorders in family members were asked in the questionnaires.

## Sample collection

Vaginal samples were collected using sterile cotton swabs and were conserved in phosphate buffer saline (PBS) at -70°C until tested. Blood samples were collected in 5-ml volumes and the sera were separated by centrifugation at 2500 rpm (1090×g). All the sera were aliquoted into several tubes to avoid excessive freeze-thaw cycles and were stored at -20°C prior to analysis.

## DNA extraction

We used boiling method to extract DNA from vaginal samples, since it has been reported as a rapid and cost- effective method with a high DNA efficiency (11). Briefly, after removing vaginal swabs from Falcon centrifuge tubes (Aratebfan, Tehran), the remaining PBS solution was centrifuged at 2000 rpm (700×g) for 15 minutes. The supernatant was then discarded and the pellet was vortexed and transferred to a 1.5 ml micro-tube. To fully remove the PBS, the micro-tube was also micro-centrifuged at 2000 rpm (295×g) for 15 minutes. After draining the supernatant fluid from the tubes, 400 µl of Tris base-EDTA (TE) buffer containing 1 mol l-1 Tris base (pH=8.0) and 0.5 mol l-1 EDTA was added to each sample. The suspension was boiled in a water bath for 10 minutes and then centrifuged at 10000 rpm (7378×g) for 10 minutes. Subsequently, the supernatant containing extracted DNA was harvested and stored at -20°C.

## *Beta-globin* polymerase chain reaction

The presence of human cells and the absence of inhibitory elements in the extracted DNA were evaluated by amplification of a 268-bp fragment of the *beta-globin* gene. Primers used in this step were:

PCO4: 5'-CAACTTCATCCACGTTCACC-3'
GH20: 5'-GAAGAGCCAAGGACAGGTAC-3' (11).

PCR was carried out on 2 µl of the extracted DNA samples in a 25 µl reaction volume consist of 20 pmol of each primer, 2 mM MgCl_2_, 0.3 mM dNTP and 1 U of Taq DNA polymerase. All PCR reagents were purchased from Cinna Gene Company (Tehran, Iran). The PCR protocol was as follows: an initial step 10 minutes at 95°C; 30 cycles of 1 minute at 94°C, 1 minute at 58°C, and 1 minute at 72°C; and a final step 8 minutes at 72°C.

## *C. trachomatis* plasmid polymerase chain reaction

To detect *Chlamydia trachomatis* in the validated DNA samples, a 241-bp fragment of chlamydial cryptic plasmid was amplified. Relevant primers for this PCR were:

KL1: 5'-TCCGGAGCGAGTTACGAAGA-3'
KL2: 5'-AATCAATGCCCGGGATTGGT-3' (12).

PCR was performed on a final volume of 25 µl containing 5 µl DNA, 6 pmol of each primer (Genfanavaran, Iran), 3 mM MgCl_2_, 0.2 mM dNTP and 1 U of Taq DNA polymerase were used for each experiment. The PCR protocol was as follows: an initial step 2 minutes at 95°C; 30 cycles of 30 seconds at 95°C, 30 seconds at 58.3°C, and 30 seconds at 72°C; and a final step 5 minutes at 72°C.

## Serological assays

Serum samples were tested by MOMP-based ELISA kits (Euroimmun, Germany) to detect anti-CT IgA and IgG antibodies. All steps were performed according to the manufacturer’s instructions. The IgA kit used in this experiment had 100% sensitivity and 97.4% specificity and the IgG kit had 78.2% sensitivity and 97.1% specificity. There was no cross reactivity with other *Chlamydia pneumoniae* positive samples for the kits.

## Statistical analysis

This was a case-control study and data analysis was carried out using GraphPad Prism version 6.07 for Windows. The Chi-square and Fishers exact tests were used for analysing diagnostic findings (PCR, IgA and IgG). Student’s t test was used to determine the mean and the standard deviations for comparing ages among participants with and without miscarriage. P<0.05 were considered statistically significant.

## Results

PCR and ELISA were performed on vaginal swabs and blood samples of 157 participants, respectively. Then the relationship between the number of previous miscarriages and the prevalence of CT infection was evaluated. The number of miscarriages was given under three categories (0, 1-2, and = 3 miscarriages).

## Detection of *Chlamydia trachomatis* by polymerase chain reaction

**Fig.1:**
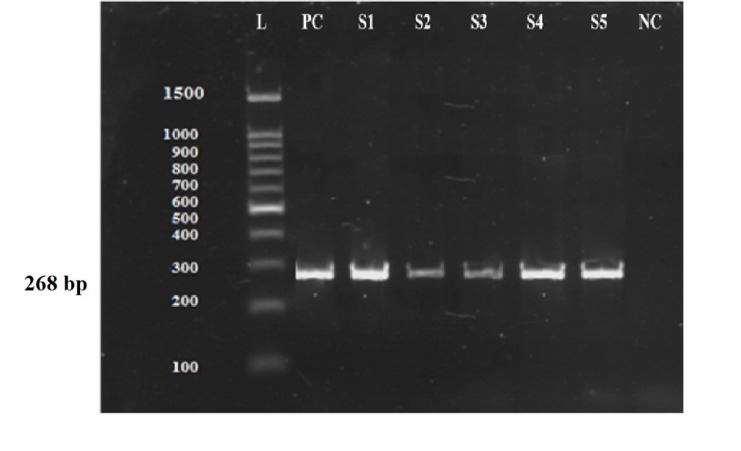
Human beta globin polymerase chain reaction (PCR) as internal control. Gel electrophoresis of amplified human *beta globin* gene presenting 268 bp amplicons in 157 extracted samples. L; 100 bp ladder, PC; Positive control, S1-S5; Samples, and NC; Negative control.

**Fig.2:**
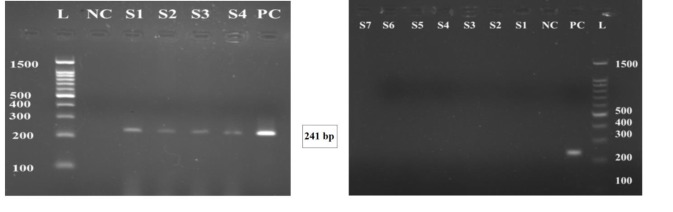
Chlamydia trachomatis (CT) plasmid polymerase chain reaction (PCR). Gel electrophoresis of amplified CT plasmid presenting 241 bp amplicons. The gel electrophoresis results on the left show the presence of CT infection in women in the miscarriage group. The gel electrophoresis results on the right present the absence of CT infection in the participants. L; 100 bp ladder, PC; Positive control, S1-S6; Samples, and NC; Negative control.

Internal control PCR showed that all samples were free of inhibitory elements (Fig .1). In both miscarriage groups together 11.3% of the patients were positive for CT infection, where all of the 60 women in the control group were tested negative (Fig .2). Detailed data with statistical analysis are shown in Table 1.

**Table 1:** The number of C. trachomatis positive cases in the control and both miscarriage groups together


Diagnostic tools	Miscarriage group n=97	Control group n=60	P value

PCR+	11	0	0.007
IgG+	4	1	0.649
IgA+	2	4	0.203


The results of the relationship between the number of previous miscarriages and prevalence of CT infection are shown in Table 2. According to the PCR data, none of the participants without a history of miscarriage were positive for CT infection. On the other hand, 5 out of 55 women with 1-2 miscarriages and 6 out of 42 women with three or more miscarriages were positive for CT as indicated by PCR. The difference between these three categories was statistically significant.

**Table 2:** Results reported for C. trachomatis infection by three diagnostic tools in women regarding their history of previous miscarriages


Number of miscarriages	Count	PCR^+^ (%)	IgG^+^ (%)	IgA^+^ (%)

0^*^	60	0	1.7	6.7
1-2	55	9.1	5.4	0
≥3	42	14.3	2.4	4.8
P value^**^		0.004	0.744	0.494


Three out of 38 women (7.9%) in =25-year age group, 4 out of 97 women (4.1%) in 26-35-year age group, and four out of 22 women (18%) in 36-45-year age group were positive for CT by PCR. Evaluation of association of mother’s age with CT infection revealed that there was a significantly higher correlation between 36-45-year age group and CT infection compared to other age groups, as indicated by PCR (P=0.042).

### Detection of *Chlamydia trachomatis* in women by ELISA IgA and IgG

In the miscarriage groups, 4.1 and 2.1% of women were positive for CT IgG and IgA antibodies, respectively. However, in the control group, these ratios were 1.7 and 6.7% of the cases (Table 1). The statistical analysis did not show any significant relationship between miscarriage and the detection of IgG or IgA chlamydial antibodies compared to the control group.

The relationship between the number of previous miscarriages and the prevalence of anti-CT antibodies was evaluated by ELISA IgA and IgG as well (Table 2). In terms of the prevalence of CT IgA antibodies, 4 out of 60 women without miscarriage history, and 2 out of 42 women with three or more previous miscarriages were CT positive. However, CT IgA antibody was not found in the women with 1-2 miscarriages. By comparing these three categories, we did not obtain any statistical significance.

CT IgG antibodies were detected in 1 out of 60 women without miscarriage history, 3 out of 55 women with 1-2 miscarriages and 1 out of 42 women with three or more miscarriages. The difference among these three categories was not statistically significant.

## Discussion

According to our PCR results, there is a positive relationship between miscarriage and underlying CT infection. The association between molecular evidence of CT infection and miscarriage has been reported by previous studies (8, 13). Also, in another study in Australia, miscarriage was attributed to the presence of chlamydia and gonorrhoea detected by PCR before pregnancy (14). 

Furthermore, in our study a significant molecular relationship was shown between the 36-45-year age group and the incidence of chlamydia positivity, which was in agreement with other studies. In 2010 Jenab et al. (11), reported a correlation between CT infection and 35-45-year age group, in a study on asymptomatic and symptomatic women in Isfahan, Iran. In a study in West Midlands, UK, it was found that there is a remarkable increase in the rate of STIs even in older adults, aged =45 years old (15). Also, Parish et al. (16) found that CT infection is concentrated in the 25-44-year age range in China. It has been reported that in China and other Asian societies, onset of sexually transmitted diseases can be late due to sexual activity beginning after reaching adolescence. Nonetheless, it has been observed in some researches that CT infection is more frequent in younger ages (17, 18). To the best of our knowledge, there is not a very clear reason for the incidence of CT infection in older ages. 

Our ELISA results showed no significant relationship between the number of previous miscarriages and CT infection, which was in accordance with earlier serologic studies on women suffering from recurrent spontaneous abortion (19-21). However, in two other studies it was reported that there was an association between experienced miscarriages and IgG antibodies to CT (7, 22). We observed low prevalence of CT IgG and IgA antibodies in this studied population. Its reason might be CT serotype replacement with fewer immunogenic types leading to lower antibody levels over time (23). Likewise, a 20-year long timed study in Finland showed decreased CT sero-prevalence and increased current infection prevalence detected by nucleic acid amplification tests over time (24). The reason can be reinfection due to untreated sex partner. In addition, immunity to CT is serovar-specific, partial and short-term (25), which can raise the rate of acute infection in women.

Despite the accuracy of the tests, the CT-positive samples were surprisingly confirmed by only one of our three diagnostic tools (PCR, ELISA IgG and ELISA IgA). For example, all PCR-positive samples were IgG/IgA-negative or IgG-positive samples were PCR/IgA-negative. This contradiction may happen due to different reasons. Positive serologic and negative molecular detection of CT may be due to an old infection or resolution of CT (26, 27) or relocation of CT from the lower to the upper genital tract (28, 29). Moreover, positive molecular and negative serologic detection of CT can be due to further lower genital infection, very low organism concentration in the upper genital tract and below the immune system detection level (30), delayed or even absent CT humoral responses in serum in spite of clinical symptoms (31, 32), early antibiotic consumption leading to persistent or chronic infection before recognizing the bacterium by the immune system (arrested immunity) (33), decrease in the anti-CT antibodies titre below the ELISA detection level (34), or primary infection. Also, in another study there were pregnant women who were positive for endo-cervical CT IgA, but negative for CT DNA, possibly due to a recently cleared CT infection, upper genital infection or a positive cervical serology caused by blood contamination containing CT antibodies (35). Therefore, IgA antibodies do not indicate recent CT infection. Instead, according to several studies, they have been attributed to chronic or persistent CT infections (28). 

At present, these alternative explanations for the discrepancy between molecular and serological results are not evident at this point, as they are case-dependent. This reflects the unique adaptive immunity in the genital tract compared to other mucosal sites. There is an association between specific host immune responses and susceptibility to or protection from CT infection (36). In fact, individual’s immune system defines that CT resolves, enters the resistance phase or reinfection occurs. The pregnancy itself is a reason of changes in the host immune responses (37). Also, person-to-person variation in responding to CT infection is due to the women's genital tract specific microbiota (38). Perhaps a long-term follow up with a larger number of participants will lead to more definitive explanations for the discrepancy between the test results.

## Conclusion

Taken together, to improve the precision and the efficiency of chlamydia detection in the current CT screening tests in clinical laboratories (usually ELISA), it is recommended that molecular tests, such as PCR, be performed as gold standard tests. Moreover, serological tests are helpful in evaluating disease conditions to differentiate ongoing from past damages caused by CT. In cases of past CT infections or upper genital infections not amenable to sampling, a serological test is an effective method to detect the infection and its importance has not faded. However, PCR is the test of choice to detect current CT infection, CT infection at the earliest days of transmission, and persistent CT infection in arrested-immunity cases. Thus, the inclusion of CT molecular and serological screening tests to other pregnancy and prenatal tests, which could allow for early detection and treatment of this infection, would decrease adverse reproductive outcomes.
